# Supplementary material for: Effects of Dandelion Extract on Promoting Production Performance and Reducing Mammary Oxidative Stress in Dairy Cows Fed High-Concentrate Diet
Source: Int J Mol Sci. 2024 May 31;25(11):6075. doi: 10.3390/ijms25116075 (PMC11172500; doi:10.3390/ijms25116075)
Supplement: Supplementary file 1 [file ijms-25-06075-s001.zip › ijms-2990037-supplementary.pdf]

Effects of dandelion extract in promoting production performance and reducing mammary oxidative stress in dairy cows fed high-concentrate diet

Yan Zhang, Musa Mgeni, Ziqing Xiu, Yu Chen, Juncai Chen, Yawang Sun*

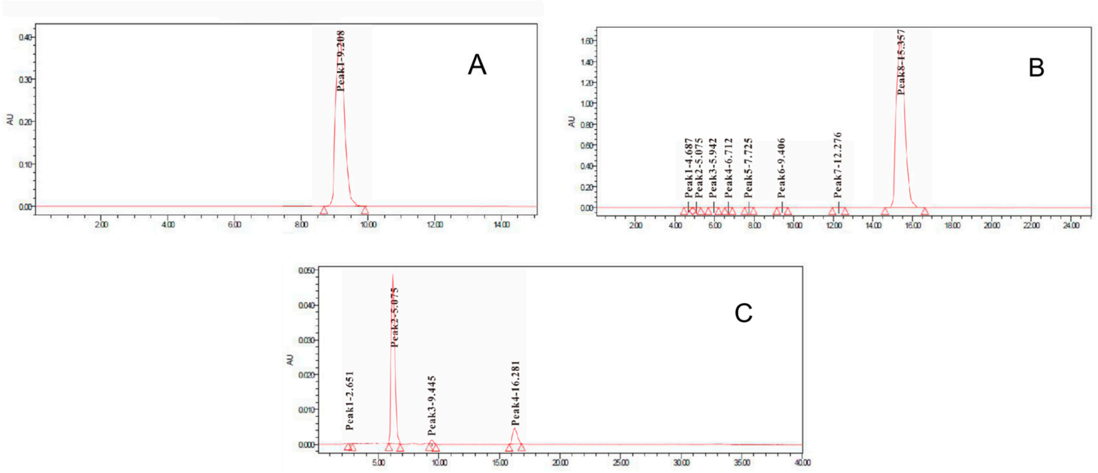

Figure S1. The high performance liquid chromatography (HPLC) chromatograms for measuring the contents of Isorhamnetin-3-o-glucoside and quercetin in dandelion aqueous extract (DAE). (A) Standard Isorhamnetin-3-o-glucoside; (B) Standard quercetin; (C) Isorhamnetin-3-o-glucoside and quercetin in DAE.
